# Statin Use and the Risk of Hepatocellular Carcinoma: A Meta-Analysis of Observational Studies

**DOI:** 10.3390/cancers12030671

**Published:** 2020-03-13

**Authors:** Md. Mohaimenul Islam, Tahmina Nasrin Poly, Bruno Andreas Walther, Hsuan-Chia Yang, Yu-Chuan (Jack) Li

**Affiliations:** 1 Graduate Institute of Biomedical Informatics, College of Medical Science and Technology, Taipei Medical University, Taipei 110, Taiwan; d610106004@tmu.edu.tw (M.M.I.); d610108004@tmu.edu.tw (T.N.P.); itpharmacist@gmail.com (H.-C.Y.); 2 International Center for Health Information Technology (ICHIT), Taipei Medical University, Taipei 110, Taiwan; 3 Research Center of Big Data and Meta-analysis, Wan Fang Hospital, Taipei Medical University, Taipei 110, Taiwan; 4 Department of Biological Sciences, National Sun Yat-Sen University, Kaohsiung City 80424, Taiwan; bawalther2009@gmail.com; 5 Department of Dermatology, Wan Fang Hospital, Taipei 11031, Taiwan; 6 TMU Research Center of Cancer Translational Medicine, Taipei Medical University, Taipei 11031, Taiwan

**Keywords:** Hepatocellular carcinoma, liver cancer, liver cirrhosis, fatty liver, liver fibrosis, statins

## Abstract

*Background and Aims*: Statins are the first-line medication to treating hypercholesterolemia. Several studies have investigated the impact of statins on the risk of hepatocellular carcinoma (HCC). However, the extent to which statins may prevent HCC remains uncertain. Therefore, we performed a meta-analysis of relevant studies to quantify the magnitude of the association between statins use and the risk of HCC. *Methods:* A systematic literature search of PubMed, EMBASE, Google Scholar, Web of Science, and Scopus was performed for studies published between January 1, 1990, and September 1, 2019, with no restriction of language. Two reviewers independently evaluated the literature and included observational and experimental studies that reported the association between statin use and HCC risk. The random-effect model was used to calculate the overall risk ratio (RR) with a 95% confidence interval (CI), and the heterogeneity among the studies was assessed using the *Q* statistic and *I*^2^ statistic. The Newcastle Ottawa Scale (NOS) was also used to evaluate the quality of the included studies. *Results:* A total of 24 studies with 59,073 HCC patients was identified. Statin use was associated with a reduced risk of HCC development (RR: 0.54, 95% CI: 0.47–0.61, *I*^2^ = 84.39%) compared with nonusers. Moreover, the rate of HCC reduction was also significant among patients with diabetes (RR: 0.44, 95% CI: 0.28–0.70), liver cirrhosis (RR: 0.36, 95% CI: 0.30–0.42), and antiviral therapy (RR: 0.21, 95% CI: 0.08–0.59) compared with nonusers. *Conclusion*: This study serves as additional evidence supporting the beneficial inhibitory effect of statins on HCC incidence. The subgroup analyses of this study also highlight that statins are significantly associated with a reduced risk of HCC and may help to direct future prevention efforts. Additional large clinical studies are needed to determine whether statins are associated with a lower risk of HCC.

## 1. Introduction

Hepatocellular carcinoma (HCC) is a growing public health issue worldwide and the most common primary malignancy of the liver [1]. HCC has received close attention for being the sixth most frequent type of cancer and the second leading cause of cancer-related mortality worldwide [2]. The incidence rate of HCC patients has increased significantly, predicted to rise to 22 million by 2032 [3]. HCC often develops in patients with chronic liver disease [4]. Chronic liver diseases, such as hepatitis C virus (HCV), hepatitis B virus, nonalcoholic fatty liver disease, autoimmune liver disease, and alcoholic liver disease lead to liver cirrhosis and eventually to HCC [5,6,7]. Liver cirrhosis is present in approximately 98% of HCC patients [8]. Previous studies also reported that patients with diabetes had a greater (two-fold) risk of HCC incidence than those without diabetes [4,9]. Several studies have reported an inverse association between statin use and chronic liver diseases [10,11], including HCC development [12]. 

**Figure 1:**
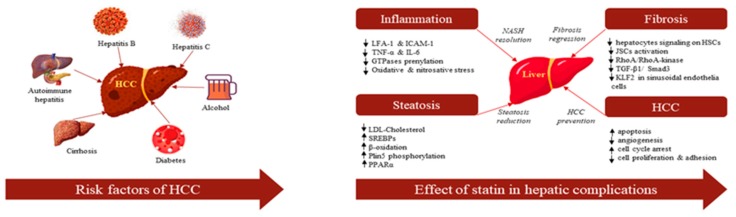
Risk factors of hepatocellular carcinoma (HCC) and the effect of statins in hepatic diseases.

Statins, 3-hydroxy-3-methylglutaryl coenzyme A (HMG-CoA) reductase inhibitors, are commonly used as standard therapy for the management and prevention of cardiovascular disease and stroke by reducing blood cholesterol level [13]. A meta-analysis of ten epidemiological studies involving 4298 patients with HCC demonstrated that statin use reduced HCC incidence (Odd Ratio (OR): 0.63, 95% confidence interval (CI): 0.52–0.76) compared to nonusers [14]. Besides their efficiency in cholesterol reduction in vitro preclinical studies showed that statins also have antiangiogenic, immunomodulatory, antiproliferative, and antifibrotic properties that probably reduce tumor growth or HCC development [15,16]. Moreover, in vivo studies showed promising results in antitumor effects such as the inhibition of cell proliferation and the promotion of tumor cell differentiation in various animal models [17,18]. Statins perhaps help to prevent HCC by suppressing oncogenic pathways including *Rho*-dependent kinase [19], tumor necrosis factor (*TNF*)-mediated interleukin (*IL6*) production [20], *Akt* [21], Myc-medicated cell proliferation, and so on [22] (Figure 1). 

The beneficial effects of statin use have ubiquitously been reported for liver cancer patients. Therefore, it is important to evaluate their effects in various doses, types, regions, and other disease conditions. To better evaluate the extent to which statins may reduce the risk of HCC, we surveyed recently published relevant studies that investigated the association between statin use and the risk of HCC development. Our primary objective was to resolve discrepancies and to measure the nature and magnitude of the association between statins and the risk of HCC development.

## 2. Results

### 2.1. Literature Screening 

**Figure 2:**
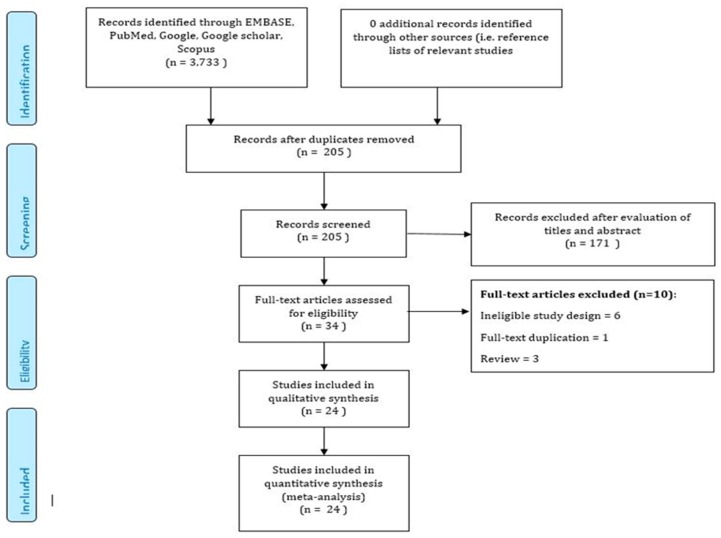
PRISMA (Preferred Reporting Items for Systematic Reviews and Meta-Analyses) flow diagram for study selection.

The initial publications search of the electronic databases yielded 3245 publications. After eliminating duplication, a total of 3113 studies were excluded based on the predefined exclusion criteria which left 32 articles for full-text review. Furthermore, two articles were added after screening the reference lists of the 32 relevant articles. Based on the review criteria, another 10 articles were excluded, which left a total of 24 publications for our present meta-analysis [23,24,25,26,27,28,29,30,31,32,33,34,35,36,37,38,39,40,41,42,43,44,45,46] (Figure 2). 

### 2.2. Study Characteristics

Table 1 shows a summary of the included 24 publications. The publications comprised 2,674,298 participants, with 59,073 HCC participants. Twelve publications were case-control studies [23,24,26,27,30,32,33,34,35,39,40,43], ten publications were cohort studies [23,25,28,29,31,36,37,38,41,42], and three publications were randomized control trial studies [44,45,46]. Of these, twelve publications were from Western [23,24,28,30,32,33,38,40,41,42,43,44,46] countries and twelve publications were from Asian [25,26,27,29,31,34,35,36,37,39,44,45] countries. All the studies used the International Classification of Diseases (ICD) code to identify HCC patients and the ATC (Anatomical Therapeutic Chemical Classification System) code to identify statin users. 

### 2.3. Study Quality

We utilized the Newcastle Ottawa scale to assess the quality of each study, which is usually applied for non-randomized studies and has been recommended by the Cochrane collaboration [47]. We also assessed randomized study quality with the Cochrane tools (Cochrane Community, London, UK) [48]. The range of the NOS (The Newcastle-Ottawa Scale) score was 7–9, and therefore, the level of evidence was high.

### 2.4. Meta-Analysis

#### 2.4.1. Primary Analysis

**Figure 3:**
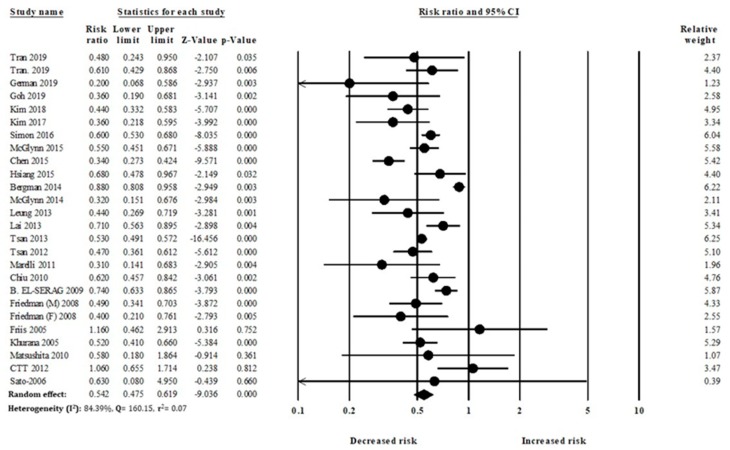
Statin use and hepatocellular carcinoma (HCC) risk.

Our meta-analysis comprised 24 studies with 59,073 HCC individuals. In the pooled analysis, overall statin use was significantly associated with a reduced risk of HCC development (RR: 0.54, 95% CI: 0.47–0.61), compared to nonusers (Figure 3). 

#### 2.4.2. Secondary Analysis

**Figure 4:**
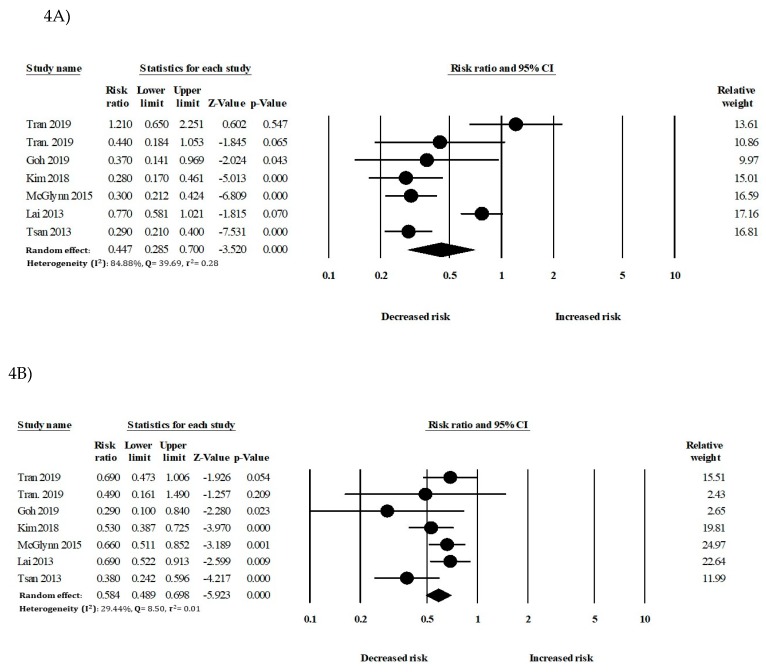
Statin use and the risk of hepatocellular carcinoma (HCC) in patients with (**A**) DM and (**B**) without diabetes mellitus (DM).

**Figure 5:**
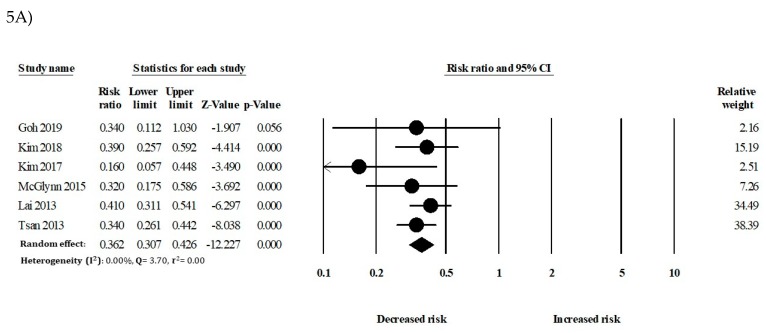

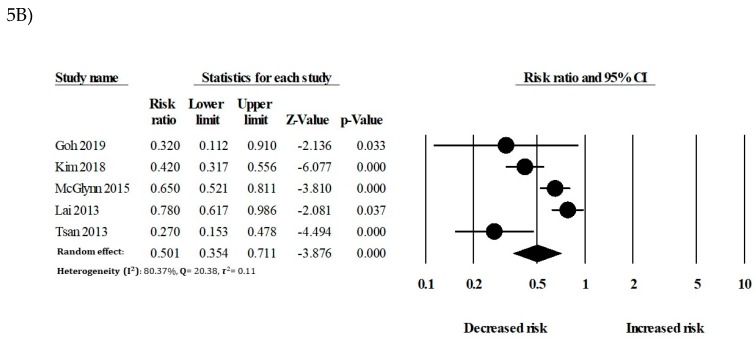
Statin use and the risk of hepatocellular carcinoma (HCC) in patients with (**A**) liver cirrhosis and (**B**) without liver cirrhosis.

In the secondary analysis, the relationship between statin use and HCC development in patients with DM (Diabetes Mellitus), liver diseases, or antiviral therapy was considered. In patients with DM, statin use was significantly associated with a reduced risk of HCC (RR: 0.44, 95% CI: 0.28–0.70) compared to that of nonusers. In patients without DM, statin use also showed a significant risk reduction of HCC (RR: 0.58, 95% CI: 0.48–0.69) (Figure 4). In patients with liver cirrhosis, statins had an inverse association with HCC (RR: 0.36, 95% CI: 0.30–0.42). In patients without liver cirrhosis, statin use also showed a significant reduction of HCC incidence (RR: 0.50, 95% CI: 0.35–0.71) (Figure 5).

### 2.5. Subgroup Analysis

The study design, region, dose, and different types of statins were considered in the subgroup analysis (Table 2). The overall pooled Risk Ratio (RR) for the observational study design was 0.52 (95% CI: 0.46–0.60), and the overall pooled RR for the RCT study design was 0.95 (95% CI: 0.61–1.47). Furthermore, we investigated the association between statin use and HCC development among patients from different regions. The pooled RR for Asian and Western populations were 0.49 (95% CI: 0.42–0.57), and 0.59 (95% CI: 0.49–0.70), respectively. 

In the dose-dependent analysis, risk reduction was accentuated with an increase of the cumulative defined daily doses (cDDD) compared with nonusers (RR 0.55 (95% CI: 0.46–0.65) and RR 0.47 (95% CI: 0.36–0.61) for ≤365 cDDD and >365 cDDD, respectively; *p* for trend <0.0001). An analysis of different types of statins showed protective effects, particularly for fluvastatin (RR 0.41, 95% CI: 0.25–0.66, *p* < 0.001), lovastatin (RR 0.43, 95% CI: 0.21–0.86, *p* = 0.01), rosuvastatin (RR 0.47, 95% CI: 0.26–0.84, *p* = 0.01), simvastatin (RR 0.54, 95% CI:0.46–0.63, *p* < 0.001), and atorvastatin (RR 0.55, 95% CI: 0.43–0.69, *p* < 0.001). However, cerivastatin and pravastatin showed protective effects (RR 0.61, 95% CI: 0.26–1.42, *p* = 0.25 and RR 0.76, 95% CI: 0.56–1.03, *p* = 0.08) but were statistically insignificant (Supplementary Figure S1–12).

### 2.6. Publication Bias

To detect publication bias, the pooled analysis or subgroup analyses were not sufficient. Therefore, we used the Egger test and the Begg test to identify publication bias. However, visual inspection of the funnel plot revealed no publication bias, later confirmed by the Begg adjusted rank correlation test (Supplementary Figure S13).

## 3. Discussion

### 3.1. Major Findings

In this meta-analysis of 24 studies with a total of 59,073 HCC individuals, statins were significantly associated with reductions in the risk of HCC (RR: 0.54, 95% CI: 0.47–0.61, *I*^2^ = 84.39%). We also analyzed the beneficial effect of statins on HCC risk reduction in patients with high-risk factors (Supplementary Figure S14), including diabetes and liver cirrhosis. Previous studies reported that patients with diabetes and liver cirrhosis were significantly associated with an increased risk of HCC. However, findings of our study showed that statins significantly reduced the risk of HCC in patients with diabetes and liver cirrhosis. Moreover, the rate of HCC reduction with statins use was greater in patients with diabetes or liver cirrhosis than in patients without diabetes or liver cirrhosis. Higher cumulative doses of statins use were associated with greater risk reductions than lower cumulative doses of statins. The use of fluvastatin, lovastatin, and rosuvastatin showed greater effects for reducing the risk of HCC than the use of other statins. 

### 3.2. Comparison with Other Studies

The findings of this study on HCC risk are similar to three previous systematic reviews and meta-analyses. A study in 2013 found that statins decreased HCC in analyses of 10 studies with a total of 1,459,417 participants with 4298 HCC patients (OR: 0.63, 95% CI: 0.52–0.76) [14]. In the next year, a meta-analysis that included 12 studies with a total of 5,640,313 participants also suggested a reduction of HCC (RR: 0.58, 95% CI: 0.51–0.67) [49]. Another meta-analysis of five observational studies with 87,127 participants also evaluated different types of statin use and reduction of HCC risk [50] and found that fluvastatin is the most effective for reducing HCC risk (RR: 0.55, 95% CI: 0.26–1.11) compared with the reductions associated with other types of statins. Our study has updated and extended the evidence of these prior systematic reviews and meta-analyses in three ways. First, we included more observational studies from different continents. Second, we included more subgroup analyses than previous studies. Finally, we evaluated several additional factors associated with statins use and HCC risk, e.g., diabetes and liver cirrhosis, to examine any possible bias or influence of these additional factors. 

### 3.3. Biological Plausibility

**Figure 6:**
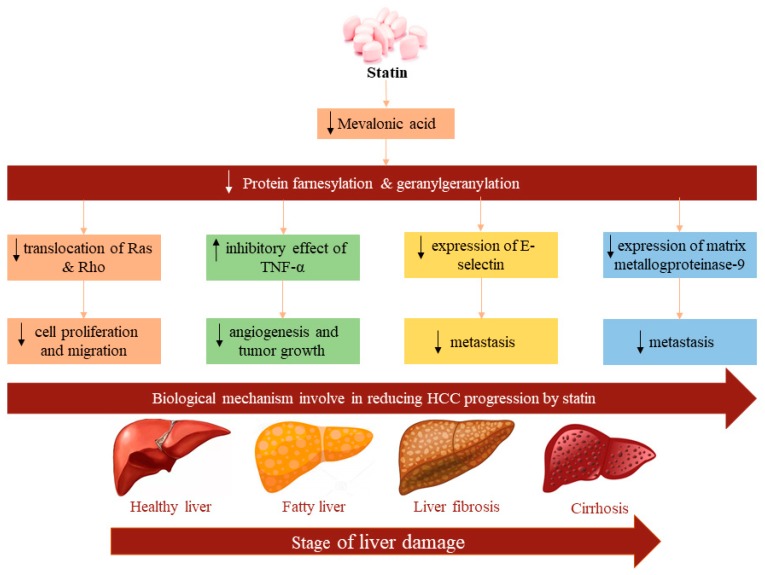
Mechanism of statins to prevent hepatocellular carcinoma (HCC).

There are several convincing biological explanations that statin use can reduce the risk of HCC. Statins indeed have several pleiotropic effects to reduce the risk of HCC, including antioxidative, anti-inflammatory, endothelial function, and anti-fibrotic properties (Figure 6). Statins are currently the ultimate choice to treat hypercholesterolemia because they inhibit the mevalonate pathway which is mainly responsible for stimulating cholesterol synthesis [51]. However, the use of statins actually reduces the expression of downstream metabolites of the cholesterol synthesis pathway, including farnesyl pyrophosphate, and geranyl pyrophosphate, thereby slowing the prenylation of *GTPase* down, which decreases the translocation of *Ras* and *Rho* and their functions, decreasing cell proliferation and migration [52]. Steatosis-induced *HSC* activation is suppressed by statins, through the paracrine effect of hepatocytes. Statins can also downregulate profibrogenic gene expressions (*TGF-β1*, *α-SMA*, and tissue inhibitor of metalloproteinases-1) and protein expression of *αSMA* in *HSCs*; thus, it reduces liver fibrosis [53]. Furthermore, chronic hepatic injury is also an important factor in HCC development [54]. Statin use further induces hepatocyte apoptosis by secreting inflammation-mediated damage occurring molecular patterns including *IL-6, IL-1β, TNF*, and reactive oxygen species (ROS) [55]. However, statins have the ability to mitigate hepatic inflammation by deactivating *IL-6* and *TNF-α* expression and by significantly attenuating metalloproteinases activity and production of ROS [53,56]. Intratumor heterogeneity supplies energy for tumor evolution and drug resistance [57,58]. It is both intrinsically timely and important to properly address the current use of statins and to provide an outlook for future use, where statins may be applied to a wider range of HCC reduction [59,60]. Finally, statins activate the protooncogenic transcription factor *Myc* [61] and accelerate the expression of the suppressor *miRNA-145* [62], which ultimately controls tumor cell migration and invasion. 

### 3.4. Quality of the Evidence

This study is a large updated meta-analysis on this topic, including both randomized controlled trials and observational studies (case-control and cohort studies). All the included studies were of higher quality with their respective NOS scores ranging between 7 and 9. All the effect sizes ware adjusted with potential confounding factors (Supplementary Figure S15), and the heterogeneity was relatively low in both primary and subgroup analyses. 

### 3.5. Study Limitations

This meta-analysis has several limitations that need to be addressed. First, all the included studies were adjusted with potential confounding factors, but these confounding factors were not the same. For example, some failed to adjust their study for factors such as the severity of liver disease (classified with the Child–Pugh score), alcohol consumption, diabetes, or different viral diseases. Second, most included studies were observational (cohort and case-control study) and the HCC patients were identified by the ICD code. Therefore, it is not clear that the HCC patients who used statins were followed properly and what was the medication compliance of these populations. Third, some studies used the same databases to assess the association between statin use and HCC risk. Even though the study duration was not the same for any of the included studies, some patients might overlap in the included studies. 

### 3.6. Research and Clinical Implications

Our updated meta-analysis included a large number of studies with 59,073 HCC patients from diverse populations (four continents), and the pooled risk ratio was less than 0.5 with a narrow confidence interval. Our findings therefore strongly suggest that statin use has a significant and clinically relevant effect on HCC reduction, including with various conditions such as diabetes and other types of liver diseases. Recently, concern has been raised about the potential hepatotoxicity of statins, and several guidelines regarding statin use also persistently warn of these risks. However, many studies have also reported that statin-induced hepatotoxicity is extremely low (less than 3% of all patients taking statins). A significant amount of retrospective studies mentioned that statins are safe for use in patients with cirrhosis, even if statins are more effective in reducing liver decompensation and hepatocellular carcinoma. However, prescribing statins ubiquitously is not recommended in patients with all types of cirrhosis. A study by Kaplan et al. [62] suggested that patients with serious cirrhosis/advanced decompensated cirrhosis (CTP C) may experience more unwanted consequences than benefits associated with initiating or continuing statin therapy. If the patients are diagnosed with decompensated cirrhosis, then statins should be prescribed with caution at low doses and should be accompanied with timely monitoring of creatinine phosphokinase levels [63]. Furthermore, Abraldes et al. [64] reported that the use of statins may be risky in Child–Pugh B or Child–Pugh C patients whose total bilirubin levels are quite high. Patients with Child–Pugh A cirrhosis can get more benefits from statin use but there is no specific evidence for dose and duration. However, longer duration of statin use is recommended to get better beneficial effects. 

Our results support the hypothesis that statins could be used as an anticancer therapy targeting the mevalonate pathway. The current study found that lipophilic statins, such as lovastatin or simvastatin, have a higher beneficial effect on HCC than hydrophilic statins such as pravastatin. Another finding from our subgroup analyses suggested that statins showed a higher beneficial effect in the Western population compared with the Asian population. Variation in genetic structure or polymorphism between Asian and Western people can influence statins’ pharmacokinetics and pharmacodynamics properties [64]. More importantly, it showed a chemopreventive role in different kinds of populations. According to these findings, a prospective evaluation should be carried out on whether statins can be used as anticancer drugs. The chemopreventive effects of statins have only been shown in observational studies, but the pooled estimate of three RCTs did not show a significant chemopreventive effect. However, these studies included only a small number of individuals (81 participants) and shorter duration of follow-up. It is also important to address that the primary outcome of these RCTs focused on the effect of statins on cardiovascular mortality. Moreover, the patients included in these RCTs had at low risk for development of HCC. Therefore, these studies were not well designed to distinguish significant differences in the two groups (placebo vs statin) with regard to HCC development. They did not screen the HCC patients because the incidence of HCC was the secondary outcome. Thus, post hoc analysis of these trials was not to judge the protective effect of statins against HCC. To make this issue clear, additional prospective randomized controlled trials are warranted to justify the findings from our primary and subgroup analyses. 

However, two large clinical trials are ongoing in the United States and Taiwan which have immense potential to justify or nullify our findings in our updated meta-analysis. In the prospective randomized control trial Phase-II (*NCT02968810*) of the United States study, researchers are evaluating the preventive effect of statin on liver cancer in patients with liver cirrhosis. Similarly, in Taiwan, another prospective randomized clinical trial phase IV *(NCT03024684*) is evaluating preventive HCC recurrence after curative treatment. Since it takes a long time and is expensive to develop a new drug, drug repurposing is getting researchers’ attention in order to find new indications. Therefore, the results of these clinical trials and our updated meta-analysis bring new hope to patients with HCC. 

## 4. Methods

### 4.1. Meta-Analytic Guidelines

In this study, we followed the Preferred Reporting Items for Systematic Reviews and Meta-Analyses (PRISMA) flow diagram for study inclusion and exclusion. Moreover, we also considered the Meta-analysis of Observational Studies in Epidemiology (MOOSE) guidelines for observational studies [65].

### 4.2. Databases and Search Strategy

We systematically searched for relevant studies in PubMed, EMBASE, Web of Science, Google Scholar, and Scopus for studies published between January 1, 1990, and September 1, 2019, with no restriction of language. The following search terms were used to finds potential studies: “hepatocellular carcinoma”, OR “liver cancer”, and “statins” OR “simvastatin”, OR “atorvastatin”, OR “lovastatin”, OR “HMG-CoA reductase inhibitor(s)”, OR “pravastatin”, OR “rosuvastatin” OR “cerivastatin” OR “pitavastatin” (Supplementary Table S1). Our initial evaluation was separately conducted by two authors (M.M.I. and T.N.P.). They cross-checked all the reference lists from retrieved articles to find additional relevant articles. 

### 4.3. Eligibility Criteria

Eligibility was restricted to large observational (≥200 participants) and clinical trials only which investigated the association between statin use and the risk of HCC as the primary outcome. Studies were included if they met the following inclusion criteria: a) a large observational study with at least a 6-month follow-up time; b) the study reported the development of HCC in adult individuals (≥18 years old) with statin use versus non-statin use; c) the study clearly defined statin exposure and the identification of HCC in patients; and d) the study clearly estimated the risk of HCC as a hazard ratio (HR), odds ratio (OR), and risk ratio (RR) with 95% CIs. However, studies were excluded if they are only review articles, letters to the editors, or case reports. Studies including fewer participants (<200 participants) were also excluded. 

### 4.4. Selection Process

The same two authors (M.M.I. and T.N.P.) independently screened the titles and abstracts of the previously selected literature. They followed prespecified inclusion and exclusion criteria developed through the discussion with all authors to identify relevant studies. Any disagreements during that reviewing process were resolved by the consideration of the prior guidelines; any remaining conflict was then resolved by discussion with the main investigator (Y.C.L.). The same two authors (M.M.I. and T.N.P.) independently conducted the data collection process and checked for study duplication, population sizes, and date of publications. 

### 4.5. Data Extraction

The primary outcome measures were ORs and HRs with 95% CIs for the association between statin use and the risk of HCC. Two authors (M.M.I. and T.N.P.) identified and recorded the effect sizes reflecting the higher degree of adjustment variables for possible confounding factors. Unadjusted findings and the effect of statins on HCC with other potential diseases were also extracted. Other information extracted from the included studies were author names, publication years, study designs, number of participants, number of HCC patients, process of HCC patient’s identifications, statins user’s definitions, doses information, effects of different types of statins on HCC, follow-up times, settings, and regions.

### 4.6. Assessment of Bias Risk

The Newcastle Ottawa Scale (NOS) was applied for evaluating the individual quality of each study (Supplementary Table S2). The heterogeneity among the studies was calculated using the *Q* statistic and the *I*^2^ statistic. Moreover, publication bias was assessed by the funnel plot-based (Egger test and the Begg test).

### 4.7. Statistical Analysis

We performed statistical analyses with the Comprehensive Meta-analysis (CMA) software (version: 3, Biostat, Englewood, NJ, USA). The risk ratio with 95% confidence intervals (CIs) was calculated to assess outcomes, and a *p*-value of less than 0.05 was considered significant. Furthermore, the random effect model was used to pool the effect size and funnel plots were drawn to present the effect sizes visually. We only considered adjusted effect sizes reported in studies for analysis to account for confounding variables.

## 5. Conclusions

To our knowledge, this is the most extensive meta-analysis so far that evaluated the beneficial effects of statins on HCC. The findings of this meta-analysis provided additional evidence because they showed that statin use can provide a 46% risk reduction in HCC development. Furthermore, a larger mechanistic study is warranted to confirm or refute our findings.

## Figures and Tables

**Table 1:** Characteristics of included studies.

Author	Year	Country	Study Design	Study Duration	% Male	Total Pts	Total HCC	Inclusion Criteria	Exclusion Criteria	Outcome	Adjusted with DM
Tran	2019	UK	Nested CC	2000–2011	67.3	2537	434	Having the first diagnosis of primary liver cancer, including HCC and IBDC)	Participants with a cancer diagnosis prior to baseline or in the year after baseline	OR = 0.61 (95% CI: 0.42–0.86)	Yes
		UK	Prospective Cohort	2006–2010	62.6	471,851	182	ICD-10 code C22	Participants with a cancer diagnosis prior to baseline or in the year after baseline	HR: 0.48 (95% CI: 0.24–0.94)	Yes
German	2019	USA	CC	2002–2016		102	34	ICD-9	Index cases, liver masses other than HCC, and etiologies of liver diseases other than NAFLD	OR = 0.20 (95% CI: 0.07–0.60)	Yes
Goh	2019	South Korea	Retrospective cohort	2008–2012	67.6	7713	702	ICD-9	Follow-up less than 6 months, missing data on cholesterol, age < 18, and history of HCC before the index date	HR: 0.36 (95%: 0.19–0.68)	Yes
Kim	2018	S Korea	Nested CC	2002–2013	83.6	514,866	8210	ICD-10	Pts without supporting clinical codes, indicating the presence of HCC including any liver diagnostic tests (biopsy or arteriography of hepatic artery) and any treatments of the liver (hepatectomy, liver transplantation, radiofrequency ablation, arterial embolization, radiotherapy, or chemotherapy).	OR = 0.44 (95% CI: 0.33–0.58)	Yes
Kim	2017	South Korea	Nested CC	2002–2013	81.4	1374	229	ICD-10	Pts whose first antidiabetic drug was insulin and patients aged <40 years during first antidiabetic prescription	OR: 0.36 (95% CI: 0.22–0.60)	Yes
Simon	2016	USA	Cohort	2001–2014	95.2	9135	239	ICD-9	Pts with human immunodeficiency virus (HIV) and those who had a positive hepatitis B surface antigen (HBsAg), baseline cirrhosis, or HCC	HR = 0.60 (95% CI: 0.53–0.68)	Yes
Hsiang	2015	China	Retrospective cohort	2000–2012	67.9	53,513	6,883	ICD-9	Pts with HCV or HIV coinfections, missing statin prescriptions, interferon exposure, HBsAg seroclearance within 6 months of the baseline, and age < 18	HR: 0.68 (95%0.48–0.97)	Yes
Björkhem-Bergman	2014	Sweden	CC	2006–2010	52	23,964	3994	ICD-9	NR	OR = 0.88 (95%0.81–0.96)	Yes
Chen	2015	Taiwan	Cohort	2000–2008	54.9	71,824	1735	ICD-9	Gender not clear, age < 20, patients diagnosed with cancer prior to the diagnosis of HBV	HR = 0.34 (95% 0.27–0.42)	Yes
McGlynn	2014	USA	CC	1999–2010	74.4	562	94	ICD-9	Pts with HCC or any cancer before the index date	OR = 0.32 (95% CI: 0.15–0.67)	Yes
McGlynn	2015	USA	CC	1998–2011	71.6	5835	1195	ICD-9	Pts with HCC or any cancer before the index date	OR: 0.55 (95% CI: 0.45–0.69)	Yes
Lai	2013	Taiwan	CC	2000–2009	72.6	17,400	3480	ICD-9	Pts with HCC or any cancer before the index date	OR = 0.72 (95% 0.59–0.88)	Yes
Leung	2013	Taiwan	CC	2000–2008	46.3	34,205	27,364	ICD-9	Pts diagnosed with cancer before index date, follow-up less than 6 months, and having any prior record of mastectomy	HR: 0.44 (95% CI: 0.296–0.72)	Yes
Tsan	2012	Taiwan	Cohort	1997–2008	58.2	33,413	1,021	ICD-9	Previously diagnosed HCC	HR = 0.47 (95% CI: 0.36–0.61)	No
Tsan	2013	Taiwan	Cohort	1999–2010	49.2	295,887	27,883	ICD-9	Previously diagnosed HCC	HR = 0.53 (95% CI: 0.49–0.57)	Yes
Marelli	2011	USA	Cohort	1990–2009	~52.2	91,714	105	ICD-9	Pts had insufficient history in the database	HR: 0.87 (95% CI: 0.60–1.26)	Yes
Chiu	2010	Taiwan	CC	2005–2008	68.8	2332	1166	ICD-9	Pts with wrist and hip fractures and previous history of HCC	OR = 0.62 (95% CI: 0.41–0.91)	Yes
B. EL–SERAG	2009	USA	CC	1997–2002	99.0	6515	1303	ICD-9	Previous history of liver disease	OR = 0.74 (95% CI: 0.64–0.78)	Yes
Friedman	2008	USA	Cohort	1994–2003	NR	361,859	42	ICD-9	NR	HR_male_ = 0.49 (95% CI: 0.33–0.70) HR_female_ = 0.40 (95% CI: 0.21–0.75)	No
Friis	2005	Denmark	Cohort	1989–2002	56.6	334,754	171	ICD-9	Pts with a history of cancer before study entry	HR = 1.16 (95% CI: 0.46–2.92)	No
Khurana	2005	USA	CC	1997–2002	NR	480,306	409	ICD-9	NR	OR = 0.52 (95% CI: 0.41–0.66)	No
Matsushita	2010	Japan	RCT	2010	31.5	13,724	12	ICD-9	Pts with previous history of cancer	HR: 0.58 (95% CI: 0.18–1.86)	Yes
CTT	2012	Europe, Australia, North America	RCT	2012	NR	134,537	68	ICD-9	Pts with nonfatal nonmelanoma skin cancers and benign neoplasm	HR: 1.06 (95% CI: 0.65–1.70)	No
Sato	2006	Japan	RCT	1991–1995	NR	263	1	Osaka Cancer Registry	Pts who resided outside the Osaka prefecture at entry were excluded	HR: 0.63 (95% CI: 0.11–3.54)	No

Note: NR = Not reported, CC = Case-Control, RCT = Randomized Control Trial, ICD = International Classification of Diseases, Pts = Participants, OR = Odd Ratio, HR = Hazard Ratio, CI = confidence interval, HCC = Hepatocellular Carcinoma, DM = Diabetic Mellitus, IBDC: Inclusion Body Disease of Cranes, NAFLD: Non-alcoholic fatty liver disease, HCV: Hepatitis C virus, HBV: Hepatitis B virus.

**Table 2:** Subgroup analysis.

Subgroup Analysis	No. of Studies	Adjusted RR	95% CI	*p*-Value	Test of Heterogeneity			
					Q	I^2^ (%)	Tau^2^	*p*-Value
**Study design**								
Observational	21	0.52	0.46–0.60	<0.001	155.14	85.82	0.07	<0.05
Case-control	12 *	0.56	0.46–0.67	<0.001	70.44	84.38	0.07	<0.001
Cohort	10 *	0.49	0.42–0.57	<0.001	28.87	65.36	0.02	0.001
RCT	3	0.95	0.61–1.47	0.82	1.03	0	0	0.59
**Study location**								
Asian	12	0.49	0.42–0.57	<0.001	30.61	64.06	0.03	0.001
Western	12	0.59	0.49–0.70	<0.001	71.47	81.81	0.06	<0.001
**Dose**								
≤365 cDDD	8	0.55	0.46–0.65	<0.001	5.84	0	0	0.55
>365 cDDD	6	0.47	0.36–0.61	<0.001	4.51	0	0	0.47
**Type of statin**								
Atorvastatin	6	0.55	0.43–0.69	<0.001	6.74	25.84	0.02	0.24
Lovastatin	3	0.43	0.21–0.86	0.01	3.03	34.18	0.14	0.21
Cerivastatin	2	0.61	0.26–1.42	0.25	0.89	0	0	0.34
Fluvastatin	4	0.41	0.25–0.66	<0.001	1.61	0	0	0.65
Pravastatin	5	0.76	0.56–1.03	0.08	2.48	0	0	0.64
Rosuvastatin	5	0.47	0.26–0.84	0.01	5.09	21.44	0.09	0.27
Simvastatin	7	0.54	0.46–0.63	<0.001	0.85	0	0	0.99

Note: * one study contained both case-control and cohort study design. RCT = Randomized Control Trial, cDDD = cumulative defined daily doses, RR = Risk Ratio, CI = confidence interval.

## Supplementary Materials

The following are available online at https://www.mdpi.com/2072-6694/12/3/671/s1, Figure S1: Statin use and the risk of HCC in patients (case-control study), Figure S2: Statin use and the risk of HCC in patients (cohort study), Figure S3: Statin use and the risk of HCC in patients (randomized control trials), Figure S4: Statin use and the risk of HCC in patients (Asian continent), Figure S5: Statin use and the risk of HCC in patients (Western continent), Figure S6: Statin use and the risk of HCC in patients (>365cDDD), Figure S7: Atorvastatin use and the risk of HCC in patients, Figure S8: Lovastatin use and the risk of HCC in patients, Figure S9: Cerevastatin use and the risk of HCC in patients, Figure S10: Pravastatin use and the risk of HCC in patients, Figure S11: Rosuvastatin use and the risk of HCC in patients, Figure S12: Simvastatin use and the risk of HCC in patients, Figure S13: Funnel plot, Figure S14 Adjusted risk factors, Table S1: Searching words, Table S2: Methodological quality of included studies based on the Newcastle Ottawa scale.
